# Supplementary material for: Immunohistochemical Staining Properties of Osteopontin and Ki-67 in Feline Meningiomas
Source: Animals (Basel). 2024 Nov 26;14(23):3404. doi: 10.3390/ani14233404 (PMC11640386; doi:10.3390/ani14233404)
Supplement: Supplementary file 1 [file animals-14-03404-s001.zip › animals-3303824-supplementary.pdf]

Figure S1

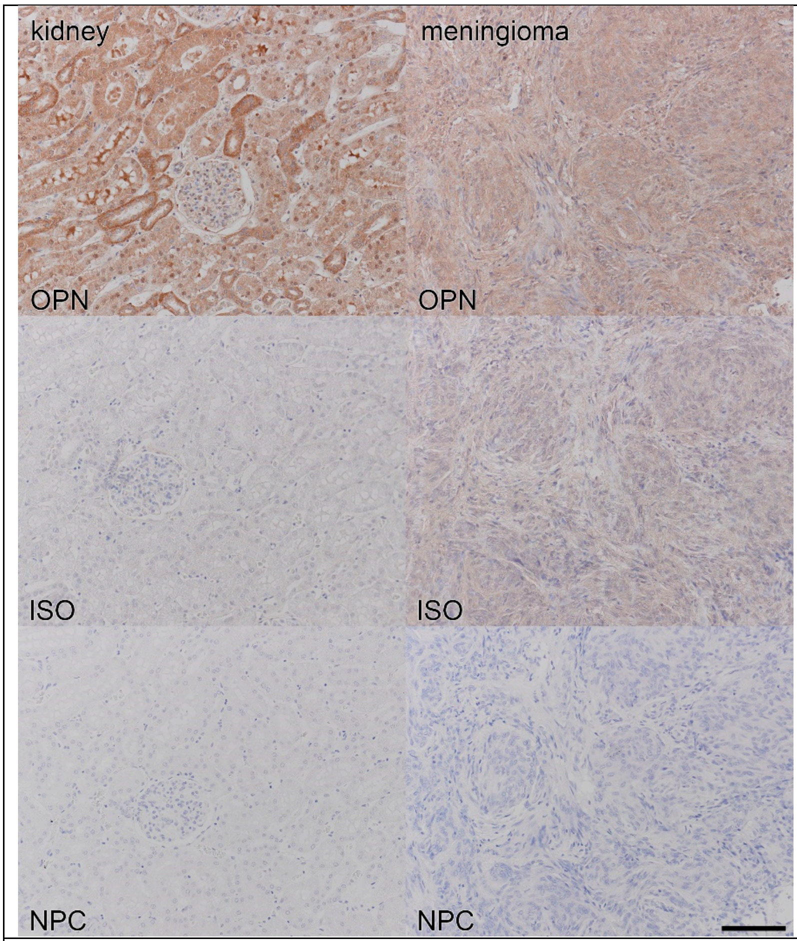

Figure S1: Immunohistochemical Controls. Positive control kidney (OPN), meningioma (OPN) and negative controls with feline tissue. ISO = rabbit isotype control, feline kidney and feline meningioma; NPC = no primary control, sections with secondary system only, feline kidney and feline meningioma. Scale bar = 100µm

Figure S2

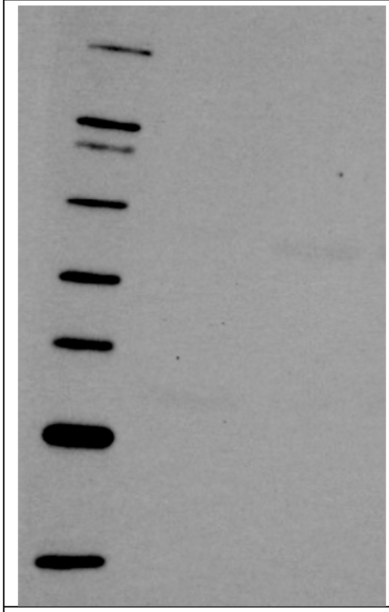

Figure S2: Western blot control. No primary control western blot with secondary antibody only. Lane 1: Molecular weight marker; Lane 2: Kidney, cat; Lane 3: Osteosarcoma, cat

Table S 1: Characteristics of 53 Patients Who underwent Meningioma Resection

Case	Breed	Age	Sex	Biopsy Sectio	Grade	Subtype	Mineralization	Cholesterol cleft	Necrosis	Micro-cystic	Ki-67 % ratio	OPN % ratio	OPNIHC	Allred
1	ESH	15	fs	B	1	fibrous-trans	+	+	+		3.95	70.28	211.00	8.00
2	ESH	16	mc	B	1	trans-meningo				+	6.76	52.38	157.00	8.00
3	ESH	11	fs	B	1	fibrous	+				27.21	73.73	221.00	8.00
4	ESH	15	mc	S	1	fibrous-trans		+			6.13	28.90	57.78	5.00
5	ESH	14	mc	S	1	fibrous	+				5.53	80.94	242.82	8.00
6	ESH	10	fs	S	1	fibrous					30.48	18.00	18.00	4.00
7	ESH	15	mc	S	1	fibrous	+		+		1.44	63.38	190.14	7.00
8	ESH	16	mc	S	1	transitional		+			10.88	14.82	14.82	4.00

9	Carthusian	13	mc	S	1	fibrous-trans					4.23	64.01	194.72	7.00
10	ESH	18	mc	S	1	fibrous-trans	+			+	12.32	56.00	167.91	7.00
11	ESH	5	ws	S	1	fibrous					5.62	20.42	40.83	5.00
12	Maine Coon	13	mc	S	1	transitional		+			6.03	1.11	1.11	2.00
13	Birman cat	15	fs	S	1	fibrous					5.35	63.46	190.40	7.00
14	ESH	8	mc	S	1	fibrous	+				7.73	36.11	72.22	6.00
15	Norwegian Forest	10	fs	B	1	transitional	+	+	+		8.01	63.50	160.49	7.00
16	ESH	5	fs	B	1	fibrous-trans	+				27.77	53.78	161.33	7.00
17	ESH	6	mc	B	1	fibrous	+				10.87	50.74	152.23	7.00
18	Maine Coon	8	mc	S	1	fibrous	+	+			7.19	24.20	48.39	5.00
19	ESH	nd	mc	S	1	fibrous	+		+		33.92	21.29	21.29	4.00
20	ESH	12	mc	S	1	fibrous					2.76	56.93	113.86	6.00
21	ESH	13	mc	S	1	Fibrous	+	+			1.64	54.46	163.38	7.00
22	ESH	10	mc	S	1	fibrous	+				3.42	25.27	75.80	6.00
23	ESH	13	mc	S	1	fibrous					11.74	52.57	157.71	7.00
24	ESH	18	mc	S	1	fibrous	+				3.94	24.07	48.15	5.00
25	ESH	10	mc	S	1	transitional		+			2.49	60.15	180.44	7.00
26	ESH	18	mc	B	1	transitional	+			+	11.32	59.66	178.97	7.00
27	ESH	15	fs	S	1	fibrous	+	+			2.16	18.56	37.13	5.00
28	ESH	16	mn	S	1	transitional	+		+		3.56	15.36	30.72	5.00
29	ESH	nd	nd	S	1	transitional	+				29.71	42.88	128.63	7.00
30	ESH	16	fs	S	1	transitional					13.25	8.05	8.05	3.00
31	ESH	16	fs	S	1	transitional					13.25	25.80	51.60	5.00
32	ESH	12	f	S	2	Fibrous-trans	+				11.42	33.20	99.60	7.00
33	ESH	11	f	S	1	transitional		+			4.09	55.70	167.11	7.00

34	ESH	8	fs	S	1	transitional		+			3.43	61.15	183.44	7.00
35	ESH	15	fs	S	1	transitional	+	+			7.77	18.25	36.53	5.00
36	ESH	11	mc	S	1	transitional			+		3.17	59.16	177.49	7.00
37	ESH	2	f	S	1	transitional	+	+			8.28	63.13	189.38	7.00
38	ESH	10	mc	B	1	transitional					11.69	50.06	150.17	7.00
39	ESH	8	mc	S	1	fibrous	+		+		6.99	45.68	91.36	6.00
40	ESH	15	fs	S	1	transitional	+	+		+	2.48	66.03	198.08	7.00
41	ESH	14	mc	S	2	Fibrous-trans	+	+		+	1.53	65.04	195.11	7.00
42	ESH	13	f	S	1	transitional			+		1.82	22.91	45.82	5.00
43	ESH	18	fs	B	1	transitional					8.38	55.77	167.32	7.00
44	ESH	16	mc	B	1	fibrous					2.07	69.43	208.30	8.00
45	ESH	19	fs	S	1	trans-meningo			+		6.64	13.32	13.32	4.00
46	ESH	13	mc	B	1	transitional	+				2.78	70.22	210.65	8.00
47	ESH	13	fs	S	1	transitional	+		+		13.05	27.37	54.75	5.00
48	ESH	18	f	S	1	transitional	+		+		47.27	23.86	47.72	5.00
49	ESH	10	mc	S	1	fibrous					2.11	77.14	231.42	8.00
50	Persian	17	f	S	1	transitional	+				9.88	34.32	102.96	7.00
51	ESH	8	fs	S	1	fibrous	+				3.54	71.68	215.05	8.00
52	ESH	14	mc	S	1	fibrous	+	+	+		4.96	28.70	86.10	6.00
53	ESH	16	mc	S	2	fibrous					2.82	23.27	46.55	5.00

Nd= no data, ESH=european shorthair cat, mc = male castrated, fs= female spayed; B=biopsy, S= section; fibrous-trans= fibrous-transitional;
trans-meningo=transitional-meningothelial;
